# Mathematical deconvolution of CAR T-cell proliferation and exhaustion from real-time killing assay data

**DOI:** 10.1098/rsif.2019.0734

**Published:** 2020-01-15

**Authors:** Prativa Sahoo, Xin Yang, Daniel Abler, Davide Maestrini, Vikram Adhikarla, David Frankhouser, Heyrim Cho, Vanessa Machuca, Dongrui Wang, Michael Barish, Margarita Gutova, Sergio Branciamore, Christine E. Brown, Russell C. Rockne

**Affiliations:** 1 Department of Computational and Quantitative Medicine, Division of Mathematical Oncology, City of Hope National Medical Center, Duarte, CA, USA; 2 Department of Hematology and Hematopoietic Cell Translation and Immuno-Oncology, City of Hope National Medical Center, Duarte, CA, USA; 3 Department of Diabetes Complications and Metabolism, City of Hope National Medical Center, Duarte, CA, USA; 4 Department of Developmental and Stem Cell Biology, Beckman Research Institute, City of Hope National Medical Center, Duarte, CA, USA; 5 Department of Mathematics, University of California, Riverside, CA, USA; 6 Mathematical and Computational Systems Biology, University of California, Irvine, CA, USA

**Keywords:** CAR T-cell therapy, mathematical modelling, antigen density, treatment efficacy, CAR T-cell dose, T-cell exhaustion

## Abstract

Chimeric antigen receptor (CAR) T-cell therapy has shown promise in the treatment of haematological cancers and is currently being investigated for solid tumours, including high-grade glioma brain tumours. There is a desperate need to quantitatively study the factors that contribute to the efficacy of CAR T-cell therapy in solid tumours. In this work, we use a mathematical model of predator–prey dynamics to explore the kinetics of CAR T-cell killing in glioma: the Chimeric Antigen Receptor T-cell treatment Response in GliOma (CARRGO) model. The model includes rates of cancer cell proliferation, CAR T-cell killing, proliferation, exhaustion, and persistence. We use patient-derived and engineered cancer cell lines with an *in vitro* real-time cell analyser to parametrize the CARRGO model. We observe that CAR T-cell dose correlates inversely with the killing rate and correlates directly with the net rate of proliferation and exhaustion. This suggests that at a lower dose of CAR T-cells, individual T-cells kill more cancer cells but become more exhausted when compared with higher doses. Furthermore, the exhaustion rate was observed to increase significantly with tumour growth rate and was dependent on level of antigen expression. The CARRGO model highlights nonlinear dynamics involved in CAR T-cell therapy and provides novel insights into the kinetics of CAR T-cell killing. The model suggests that CAR T-cell treatment may be tailored to individual tumour characteristics including tumour growth rate and antigen level to maximize therapeutic benefit.

## 1. Statement of significance

We use a mathematical model to deconvolute the nonlinear contributions of chimeric antigen receptor (CAR) T-cell proliferation and exhaustion to predict therapeutic efficacy and dependence on CAR T-cell dose and target antigen levels.

## 2. Introduction

CAR T-cell therapy is a targeted immunotherapy, demonstrating remarkable anti-tumour efficacy, particularly in the treatment of haematologic cancers [1,2]. CAR T-cell therapy is a specific type of immunotherapy where T-cells are genetically modified to recognize a tumour antigen thereby specifically redirecting T-cell cytolytic activity. Inspired by the success of CAR T-cell therapy in liquid tumours, there has been great interest in expanding the use of CAR T-cells for the treatment of solid tumours, such as glioblastoma (GBM), a highly aggressive form of primary brain cancer. Several clinical trials using CAR T-cells to treat GBM have been initiated all over the world [3–6]. At this early stage of clinical development, CAR T-cells offer much promise in solid tumours. However, the diversity of current clinical trials employing varying types of CARs for different solid tumours, target patient populations, preconditioning regimes and cell origins (autologous versus allogeneic) presents a significant challenge in identifying which aspects of a given CAR T-cell treatment protocol are most critical for its effectiveness. An additional critical challenge for CAR T-cell therapy is the potential for transient progression, where the cancer appears to progress before eventually responding to the treatment [7,8].

In order to address these challenges in CAR T-cell therapy for solid tumours, we endeavoured to study the kinetics of CAR T-cell killing with an *in vitro* system and a mathematical model. Mathematical models are useful to describe, quantify and predict multifaceted behaviour of complex systems, such as interactions between cells. A mathematical model is a formalized method to hypothesize systems dynamics, and yield solutions that predict the system's behaviour with a given set of parameters and initial conditions. Mathematical models can be versatile and tested with clinical data which may be obtained *in vivo* from non-invasive imaging [9–11] and the models can be refined when additional information about the system becomes available. Many mathematical models have been developed to understand tumour progression to guide refinement of cancer therapy regimens [12–14].

As CAR T-cell therapy is a newly advanced treatment modality, relatively few studies have used computational modelling to understand and improve this cell-based therapy. Recently, computational models have been developed to investigate cytokine release syndrome for toxicity management [15–17], effect of cytokine release syndrome on CAR T-cell proliferation [18], mechanisms of CAR T-cell activation [19,20], and dosing strategies [21]. However, it remains an open challenge how to use mathematical modelling to study and ultimately predict dynamics of CAR T-cell mediated cancer cell killing with respect to CAR T-cell dose, donor-dependent T-cell differences, cancer cell proliferation, target antigen expression, and how these factors contribute to the overall effectiveness of CAR T-cell therapy.

Based upon our pre-clinical and clinical experience with our well-characterized IL13Rα2-targeted CAR T-cell therapy for recurrent GBM [22,23], we have identified several factors which contribute to the effectiveness of CAR T-cells, namely: rates of proliferation, exhaustion, persistence and target cell killing. To study these various facets of CAR T-cell killing kinetics, we modelled the dynamics between cancer cells and CAR T-cells as a predator–prey system with a mathematical model we call CARRGO: Chimeric Antigen Receptor T-cell treatment Response in GliOma. We use a real-time cell analyser experimental system to estimate parameters of the mathematical model and then apply the model to *in vivo* human data. The long-term aim of this work is to develop a model which could be used to predict and eventually to optimize response to CAR T-cell therapy.

## 3. Methods

The CARRGO mathematical model is a variation on the classic Lotka–Volterra [24,25] predator–prey equations:

3.1 dXdt⏞cancer   cellrate   of   change=ρX(1−XK)⏞logistic   growth of   cancer   cells−κ1XY⏞CAR   T-cell induced   cancer   cell   death

and

3.2 $$\;\overset{\begin{array}{c} \mathrm{CAR}\;\;T\text{-}\mathrm{cell}\\ \mathrm{rate}\;\;\;\mathrm{of}\;\;\;\mathrm{change} \end{array}}{\overbrace{\frac{dY}{dt}}}\;=\;\underset{\begin{array}{c} \mathrm{cancer}\;\;\;\mathrm{cell}\;\;\;\mathrm{stimulated}\;\;\;\mathrm{proliferation}\\ \mathrm{or}\;\;\;\mathrm{exhaustion}\;\;\;\mathrm{of}\;\;\mathrm{CAR}\;\;T\text{-}\mathrm{cells} \end{array}}{\underbrace{\kappa_{2}XY}}-\underset{\mathrm{CAR}\;\;T\text{-}\mathrm{cell}\;\;\;\mathrm{death}}{\underbrace{\theta Y}},$$

where *X* represents the density of cancer cells, *Y* is the density of CAR T-cells, *ρ* is the net growth rate of cancer cells, *K* is the cancer cell carrying capacity, *κ*_1_ is the killing rate of the CAR T-cells, *κ*_2_ is the net rate of proliferation including exhaustion of CAR T-cells when encountered by a cancer cell and *θ* is the death rate of CAR T-cells. The parameters *ρ*, *K*, *κ*_1_, *θ* are constants and assumed to be non-negative except for *κ*_2_ which can be either positive or negative (table 1). A positive value of *κ*_2_ indicates an increased rate of CAR T-cell proliferation when stimulated by interaction with a cancer cell. A negative value of *κ*_2_ indicates exhaustion or limited activation of CAR T-cells resulting from interaction with a cancer cell. Exhaustion and hypoactivation of CAR T-cells are combined into a single value and are not modelled individually.

**Table 1.** CARRGO model parameters. All parameters are assumed to be non-negative except *κ*_2_ which may be positive or negative.

parameter	description	unit
*ρ*	cancer cell net growth rate	day^−1^
*K*	carrying capacity	cell
*κ*_1_	CAR T-cell killing rate	day^−1^ cell^−1^
*κ*_2_	net rate of proliferation and exhaustion of CAR T-cells when stimulated by cancer cells	day^−1^ cell^−1^
*θ*	CAR T-cell death rate (persistence)	day^−1^

We chose to model the net number of cancer cells and simple interactions between cancer cells and CAR T-cells because the output data from the culture system are limited to cell number over time. We therefore are only able to infer dynamics at this scale and dimension (i.e. number of cells and time). Moreover, we performed a system identifiability analysis to demonstrate the parameters of the model can be uniquely determined from the data in this experiment (see electronic supplementary material, methods) [26–29]. Future studies may examine more complex dynamics such as individual cell antigen levels, heterogeneity, resistant and sensitive sub-populations, repeated treatments, etc. with other experimental designs which directly measure these features.

### 3.1. Model assumptions

The CARRGO model treats cancer cell–CAR T-cell dynamics in this experimental condition as a closed predator–prey system. The model assumes (1) the populations are well mixed, (2) cancer cell growth is limited by space and nutrients (culture media) in the *in vitro* culture system and therefore grow logistically, (3) CAR T-cells kill cancer cells when they interact via the law of mass action, (4) the CAR T-cell killing rate does not explicitly assume a dependence on antigen density, (5) CAR T-cells may be stimulated to proliferate or to undergo loss of effector function—defined as exhaustion—upon contact with a cancer cell [30], and (6) the CAR T-cell death rate is independent of cancer cell density. We chose the logistic growth model for the cancer cell population because the fixed growth rate and carrying capacity parameters were the biological quantities of interest when comparing CAR T-cell killing kinetics across cell lines. Witzel *et al.* compared several sigmoidal growth laws including logistic, Gompertz and Richards, and showed that all these models can be fitted equally well to this form of experimental data [31]. Data supporting our model assumptions are given in electronic supplementary material, figures S1 and S2.

### 3.2. Dynamical system analysis of the CARRGO model

Closed form solutions cannot be obtained for the relatively simple CARRGO model. To study the possible dynamics of the CARRGO model, we perform classical dynamical system analysis. Detailed mathematical analysis of this model can be found in several textbooks on dynamical systems [25,32]. In the interest of informing the reader, we briefly summarize the main points here. We begin by (1) scaling (non-dimensionalizing) the variables in the system and then (2) identify stationary points and classify their stability and finally (3) interpret the stationary points and system dynamics in terms of the initial numbers of cancer cells and CAR T-cells.

First, we scale the variables in the CARRGO model to obtain a model without physical units in order to study the intrinsic dynamics of the system. We scale time, the cancer cell and CAR T-cell populations as $\tau =t\rho ,\;y=\;(\kappa_{1}/\rho )Y,\;x=X/K.$

These variables are substituted into the CARRGO model (equations (3.1) and (3.2)) to obtain the scaled dimensionless system

3.3 $$\frac{dx}{d\tau }=x(1-x)-xy$$

and

3.4 $$\begin{array}{rl} & \frac{dy}{d\tau }=\;Bxy-Ay,\\ & \mathrm{with}\;\mathrm{dimensionless}\;\mathrm{constants}\;A=\frac{\theta }{\rho },\;\;B=\frac{\kappa_{2}K}{\rho }. \end{array}\}$$

**Figure 1.**
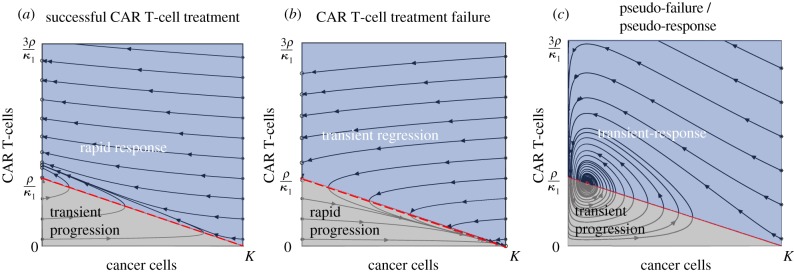
Possible dynamics from the CARRGO model. Dynamics are represented as trajectories in a two-dimensional phase-space (x,y)=(cancer cells,CAR T-cells). (*a*) Case 1: successful CAR T-cell treatment (*A* = 0, *B* = 0.2). This situation predicts all long-term dynamics to result in eradication of cancer cells with varying levels of residual CAR T-cells. (*b*) Case 2: CAR T-cell treatment failure (*A* = 0, *B* = −0.2). This situation predicts all long-term dynamics to result in cancer cells growing to carrying capacity and eventual elimination of CAR T-cells. (*c*) Case 3: pseudo-failure/pseudo-response (*A* = 0.14, *B* = 1.6). This situation predicts long-term coexistence of cancer cells and CAR T-cells, denoted P_3_ (red circle). In this situation, cancer cell and CAR T-cell populations increase, then decrease, then increase in an oscillatory manner. The dark blue regions show cancer cell response and the light grey regions show cancer cell progression. We note that all three dynamics predicted by the CARRGO model include periods of transient increase or decrease in the cancer cell population, pointing to pseudo-progression of cancer, which is a critical challenge in CAR T-cell treatment. (Online version in colour.)

The steady-state solutions of this system (equations (3.3) and (3.4)) are obtained by setting the time derivatives equal to zero. The values of the dimensionless parameters *A* and *B* determine the dynamics of the system which may be represented as trajectories in a two-dimensional phase-space of cancer cells and CAR T-cells (*x*, *y*). Three stationary points corresponding to steady-state solutions are denoted by $P_{i}=(x,y)$: $P_{1}=(0,0)$ where both the cancer cells and CAR T-cells are eliminated, $P_{2}=(1,0)$ where the cancer cell population reaches the carrying capacity and CAR T-cells are eliminated, and a coexistence of both populations, $P_{3}=(A/B,(\;1-(A/B)))$. For a given initial condition, three possible dynamics can result from this model depending on the values of *A* and *B* (figure 1).

#### 3.2.1. Case 1: successful CAR T-cell treatment (A=0,B>0)

This situation occurs when the death rate of CAR T-cells is negligible (θ≈0) relative to the proliferation rate of cancer cells (*ρ*), and the CAR T-cells are stimulated to proliferate when encountering a cancer cell $\left(\kappa_{2}>0\right)$. In this case, the equilibrium points are when the cancer cells are eliminated with some remaining CAR T-cells (0,*y*) and when the cancer cells reach carrying capacity with no remaining CAR T-cells (*K*,0). Cancer cell elimination (0,*y*) is stable only if the product of the rate of CAR T-cell killing and the number of CAR T-cells is greater than the proliferation rate of the cancer cells $\left(Y>\rho /\kappa_{1}\right)$. If the initial CAR T-cell population satisfies the inequality $Y<(\rho /\kappa_{1})-(\rho X/K\kappa_{1})$, the CARRGO model predicts a *transient progression* of cancer cells before eventual response (grey region). The point (*K*,0) is an unstable repulsor state (figure 1*a*).

#### 3.2.2. Case 2: CAR T-cell treatment failure (A=0,B<0)

This situation occurs when the proliferation rate of CAR T-cells is less than the exhaustion rate of CAR T-cells due to interaction with cancer cells. In this case, the fixed points (0,*y*) and (*K*,0) are the same as in case 1. However, the point (*K*,0) is now a stable attractor state, corresponding to the extinction of CAR T-cells and the cancer cells eventually growing to carrying capacity. Again, cancer cell elimination (0,*y*) is stable only if the CAR T-cell population is larger than the ratio of cancer cell proliferation and CAR T-cell killing rate $\left(Y>\rho /\kappa_{1}\right)$. If the initial CAR T-cell population satisfies the inequality $Y>(\rho /\kappa_{1})-(\rho X/K\kappa_{1})$, the CARRGO model predicts a *transient regression* of cancer cells before eventual rapid progression (grey region). This case is a failure of CAR T-cell treatment (figure 1*b*).

#### 3.2.3. Case 3: pseudo-failure or pseudo-response (A>0,B>0)

In this situation, the third stationary point $P_{3}$ corresponding to cancer cell and CAR T-cell coexistence lies in the first quadrant (positive numbers of cancer cells and CAR T-cells) only if A≤B. The point $P_{3}=A(B-A)/B$ is then a stable sink (figure 1*c*). This case results in an oscillating behaviour of an increase in cancer cells corresponding to tumour progression followed by a decrease in tumour cells corresponding to treatment response. The transient and oscillatory nature of these dynamics may be interpreted as a ‘pseudo’-failure and ‘pseudo’-response to the therapy. We note that cancer progression and treatment occur on finite and sometimes small time scales and therefore oscillatory dynamics may not be observed *in vivo* due to insufficient time to observe these changes.

### 3.3. Cell lines

Low-passage primary brain tumour (PBT) lines were derived from GBM patients that had undergone tumour resections at City of Hope as previously described [23,33]. Fibrosarcoma line HT1080 was obtained from the American Tissue Culture Collection (ATCC) and maintained according to recommendations. The cell line PBT030 endogenously expresses high level of IL13Rα2. HT1080 and PBT138 do not express IL13Rα2 and were lentivirally engineered to express varied levels based on different promoter strengths to investigate the relationship between killing kinetics and antigen expression level: high (greater than 70%^+^) driven by the EF1α promoter, medium (between 40%^+^ and 70%^+^) driven by the PGK promoter, low (less than 20%^+^) driven by the attenuated PGK100 promoter [34,35]. These cell lines are denoted with H, M, L, respectively (e.g. HT1080-H). These tumour cell lines were selected because they differ in aggressiveness (proliferation rates) and antigen expression levels (endogenous or engineered).

CAR T-cells were derived from healthy donor CD62 L+CD45RO+ central memory T-cell population and lentivirally transduced with second-generation IL13Rα2-targeting CARs: IL13BBζ or IL1328ζ [23,33,36,37]. Transduced product was enriched for CAR and expanded in X-Vivo media with 10% fetal bovine serum until 17 days in culture and cryopreserved. Non-transduced T-cells expanded under the same condition were used as mock control.

### 3.4. Experimental design

**Figure 2.**
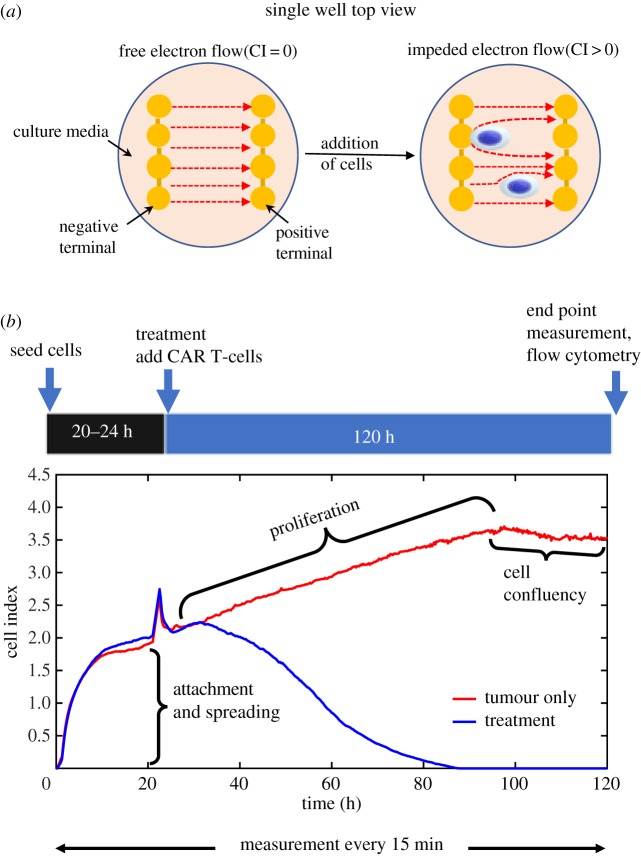
Schematic of experiment design and output data from the xCELLigence system. (*a*) The xCELLigence system for real-time monitoring of cancer cell growth and response to CAR T-cell therapy. (*b*) The output of xCELLigence is ‘cell index’ (CI) which is calculated from changes in electrical impedance in the culture plate over time. Cell index is strongly correlated with the number of cells in the well/plate. This system results in distinct regimes of cell growth dynamics, including an attachment phase followed by proliferation and confluency. The spike in CI signal at 24 h is noise resulting from removing the plate from the incubator to add CAR T-cells. The proliferation growth regime was used to fit the CARRGO model and quantify dynamics of CAR T-cell killing of cancer cells (blue curve) when compared with untreated growth (red curve). (Online version in colour.)

Real-time monitoring of cancer cell growth was performed by using xCELLigence cell analyser system [38]. This system uses electrical impedance to non-invasively quantify adherent cell density with a dimensionless number referred to as cell-index (CI). The cell-index read-out is strongly positively correlated with the number of cells in the well (*r*^2^ > 0.9) and can be used as a measure of cell number [31]. We therefore report CARRGO parameter values in units CI which can be translated into units per cell based on the linear relation. Real-time cytotoxicity assay was performed using xCELLigence system in disposable 96 well E-Plates. Prior to seeding, tumour cells were enzymatically single-celled and seeded at 25 × 10^3^, 12.5 × 10^3^ or 2 × 10^3^ cells per well depending on the cell line. Cells were either left untreated (triplicates per cell line) or treated with CAR T-cells at effector to target ratios (E : T) of 1 : 5, 1 : 10 and 1 : 20. CAR T-cells were added to the wells about 24 h after cancer cell seeding. Growth curves were recorded over 4 days with temporal resolution of 15 min (figure 2). Each cell line was treated with three IL13Rα2-targeted CAR T-cells: BBζ, 28ζ and mock. At the end of the experiment, flow cytometry was performed to measure the residual CAR T-cells, cancer cells and IL13Rα2 expression level. The details of cancer cell seeding and effector to target ratios used for the experiments are given in electronic supplementary material, table S1. The cancer cell dynamics of all the wells of 96 well E-plate for all cell lines are given in electronic supplementary material, figure S3.

### 3.5. CARRGO model fitting to experimental data

The first 24 h of the time series describes the process of cell attachment to the bottom of the plate (figure 2). The spatial process of cell adhesion and spreading in the well can be modelled as a reaction–diffusion process, described in electronic supplementary material, figure S4. Since we are interested in cell growth kinetics, we omitted the data from the first 24 h during the attachment process. The final time point is determined when the cells reach confluency which varies by cell line. Observed time of confluency for the PBT cell line was around 120 h from the time of seeding while HT1080 reached confluency within 80 h. The data points from CAR T-cell administration (24 h after seeding) up to 80% of maximum CI value (confluency) were used for model fitting to estimate the parameters. At greater than 80% of the maximum CI, the linear relationship between CI and cell number no longer holds [31].

Cancer cell net growth rate *ρ* and carrying capacity *K* (equation (3.1)) were computed by fitting logistic growth to untreated cancer cell time-series data. The CAR T-cell killing rate *κ*_1_, growth rate *κ*_2_ and death rate *θ* were computed by fitting the solution of the CARRGO model (equations (3.1) and (3.2)) to treated cancer cell time-series data by minimizing the root mean square error. Linear regression was used to determine the quality of model fitting (*R*^2^). All optimization computations were performed in Matlab with *fmincon*.

### 3.6. CARRGO model fitting to human data

A patient with recurrent glioma received CAR T-cells engineered for IL13Rα2 and showed complete tumour regression, which was published as a brief report by Brown *et al.* in 2016 [23]. We retrospectively collected the magnetic resonance imaging (MRI) data of this patient and calculated tumour volumes to be used to fit the CARRGO model. Three lesions were selected using the lesion labelling reported in Brown *et al.* [23]: lesions T6, T7 which responded to IL13Rα2 targeted CAR T-cells and lesion T9 which was a lesion that appeared later on which did not respond to the therapy. Tumour volumes for each lesion were estimated by manual segmentation of contrast-enhancing lesions from T1-weighted post-contrast MRIs. The number of cancer cells (CC) was estimated by calculating CC = [tumour volume (µm^3^)]/[GBM cell size (µm^3^)] with the average cell diameter assumed to be 20 µm [39]. The volume of a spherical cell is then given by $V=(4\pi /3){\left(10\;\mu m\right)}^{3}$. These relationships were used to estimate the total number of cancer cells in a tumour volume. The tumour growth rate (*ρ*) for lesions T6 and T7 was computed from two subsequent imaging time points following the first appearance of the lesion on MRI. Lesion T9 underwent surgical resection prior to CAR T-cell administration. The tumour growth rate for lesion T9 was computed from the pre-surgical MRI data. CAR T-cells were administered first directly into the tumour tissue and subsequently into the cerebrospinal fluid via the intraventricular injection. Because the CAR T-cells migrated to several tumour foci in the patient, we assumed a small fraction (5–10%) of the infused dose reached each individual lesion at each infusion. The CARRGO model was fitted to the time-series MRI-derived tumour volume data by minimizing the root mean square error for MRIs before and during CAR T-cell treatment to compute the rates of CAR T-cell killing *κ*_1_, exhaustion *κ*_2_ and death *θ*. For lesion T9, the CARRGO model was fitted to the MRI data following CAR T-cell administration which occurred after the partial surgical resection.

## 4. Results

### 4.1. Model/data fitting to *in vitro* data

**Figure 3.**
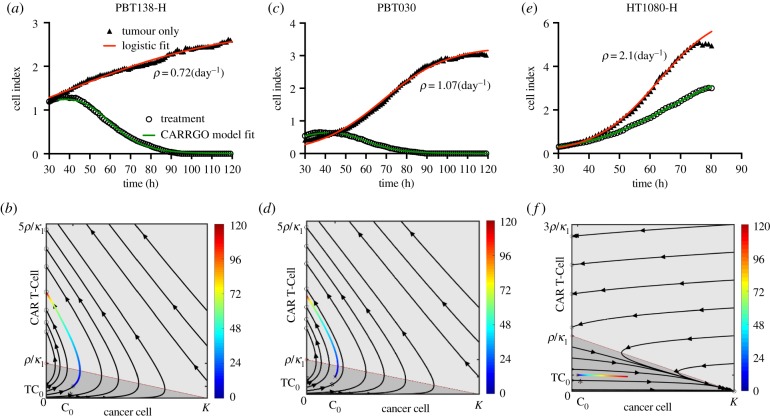
CARRGO model dynamics and *in vitro* CAR T-cell and glioma cell data. Dynamics from three cancer cell lines with and without CAR T-cell treatment along with CARRGO model fits (red line: logistic growth; green line: CARRGO model) with effector to target ratio 1 : 20. Top row: cell index from xCELLigence and model fit. Bottom row: data of treatment dynamics plotted in phase-space coloured by time (hours). (*a,b*) PBT138, tumour seeding 12.5 × 10^3^ (cells), (*c,d*) PBT030, tumour seeding 12.5 × 10^3^ (cells), (*e,f*) HT1080 high, tumour seeding 2 × 10^3^ (cells). Estimated tumour growth rate for cell line PBT138-H was *ρ* = 0.72 day^−1^, for PBT030 was *ρ* = 1.07 day^−1^ and for HT1080-H was *ρ* = 2.1 day^−1^. PBT138-H and PBT030 show successful CAR T-cell killing. HT1080-H shows CAR T-cell exhaustion and treatment failure. These dynamics are captured in the phase-space diagrams and CARRGO model parameters, which correctly capture transient progression before response for PBT138 and PBT030 (case 1, figure 1) and rapid progression for HT1080-H (case 2, figure 1). (Online version in colour.)

A high goodness of fit of the CARRGO model to the xCELLigence data was observed across all cell lines ($R^{2}=0.93\pm 0.1$, figure 3; electronic supplementary material, figure S3). To investigate the sensitivity of our model fitting to sampling frequency, we down-sampled the data by taking time intervals of 2, 5 and 10 h. No significant variation was observed in the model parameters *κ*_1_, *κ*_2_ and *θ* to the down-sampled data (repeated measure ANOVA *p* > 0.1) (electronic supplementary material, figure S5 and S6). We consistently observed very small values of the CAR T-cell death rate $\left(\theta <10^{-3}\right)$. Uniqueness of the parameters was tested by choosing 100 different combinations of values of the parameters across several orders of magnitude for the model fitting optimization procedure. We found that if the optimization converged, it converged to unique values of the parameters, which is a direct consequence of the identifiability analysis of the model and minimum number of points required to resolve the model (see electronic supplementary material, S1, figure S7 and movie S1).

### 4.2. Validation of xCELLigence dynamics with flow cytometry

Because the xCELLigence system is an indirect measure of cell number, we validated the previously reported linear relationship [38] between the cell index read-out from the machine and the number of cells measured with flow cytometry (*R*^2^ > 0.9; electronic supplementary material, S2 and figure S8). Because cell index measures the change in electrical impedance caused by both cancer cells and CAR T-cells adhering to the well, CAR T-cell dynamics are not directly measured by the system, so we compared the cancer cell (CC) to T-cell ratio (TC) from flow cytometry to that predicted from the CARRGO model. The model predicted ratio CC/TC at end time point shows a similar trend to that measured with flow cytometry, indicating the CARRGO model-predicted CAR T-cell dynamics derived from the xCELLigence data are consistent with flow cytometry measurements. This trend was observed in PBT030 and PBT138 for BBζ and 28ζ CAR T-cells and for all doses (electronic supplementary material, S2 and figure S9).

### 4.3. CAR T-cell dose-dependent dynamics

**Figure 4.**
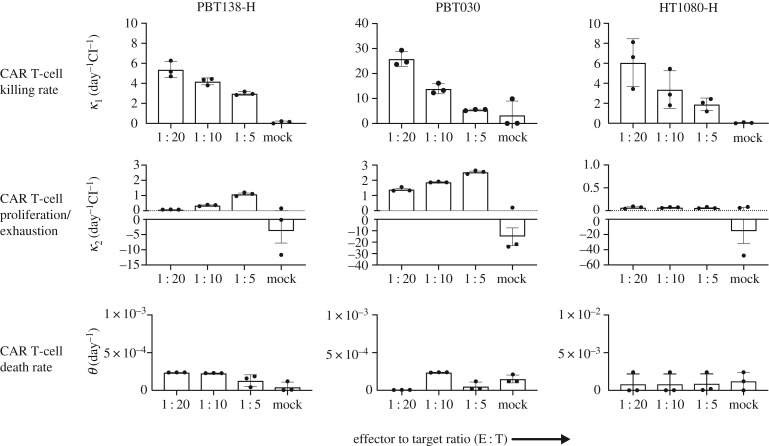
Comparisons of CARRGO model parameters with cell line and CAR T-cell dose. CARRGO model parameters: (top row) killing rate (*κ*_1_), (middle row) proliferation/exhaustion rate (*κ*_2_), (bottom row) and persistence/death rate (*θ*), for cell lines PBT138-H, PBT030 and HT1080-H treated with three different effector to target (ET) ratios (1 : 5, 1 : 10 and 1 : 20). CAR T-cell killing rate is observed to decrease with increasing ET ratio for all cell lines. This suggests CAR T-cells kill more cancer cells per unit time at a lower concentration when compared with higher ET ratio. By contrast, the CAR T-cell proliferation/exhaustion rate increases with ET ratio. This suggests that the CAR T-cells are stimulated to proliferate and are less exhausted with higher ET ratio when compared with lower. For reference, CAR T-cells are hypoactivated in mock (*κ*_2_ < 0). The CAR T-cell death rate, or persistence, is observed to be independent of target cell line and ET ratio.

We examined the effect of varying the effector to target ratio, i.e. CAR T-cell dose, for all cell lines. The CAR T-cell death rate parameter was found to be very small ($\theta <10^{-3}$ day^−1^) for all cancer cell lines and all CAR T-cells and doses. The killing rate parameter *κ*_1_ shows a negative correlation while *κ*_2_ shows positive correlation with respect to the CAR T-cell dose. The parameter *κ*_2_ was negative only for tumour line HT1080-H which indicates the exhaustion rate being much stronger than the proliferation rate of the CAR T-cells. A positive correlation of *κ*_1_ with CAR T-cell dose indicates that higher dose of CAR T-cell results in a lower killing rate, since each individual CAR T-cell has fewer number of cancer cells to encounter. The range of values for κ_1_ and κ_2_ varied with cancer cell line as the growth rate of each cell line is different from each other; however, the overall trends were preserved across the cell lines. Plots of *θ, κ*_1_ and *κ*_2_ for PBT030, PBT138 (seeding 12.5 × 10^3^) and HT1080-H treated with BBζ CAR T-cells are shown in figure 4. Other cell lines and parameters for 28ζ CAR T-cells are given in electronic supplementary material, S2 and figure S10.

### 4.4. Relating *κ*_1_, *κ*_2_ with tumour growth rate and antigen expression

**Figure 5.**
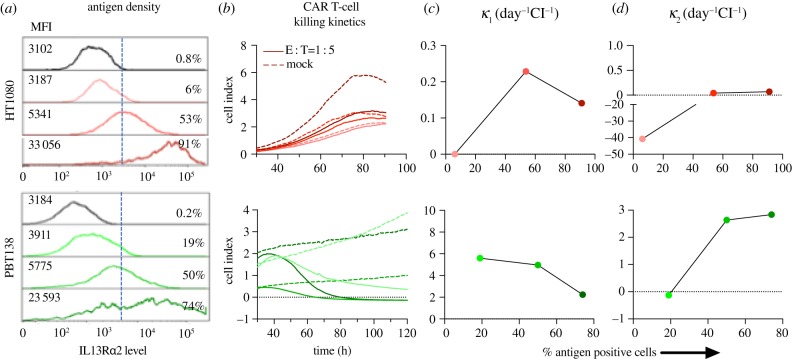
Killing kinetics of CAR T-cells when compared with antigen expression level. IL13Rα2 antigen expression measured with flow cytometry (mean fluorescence intensity (MFI), % of cells positive) for cell lines HT1080, PBT138 (mock, low, medium, high) (*a*) and CAR T-cell killing dynamics measured by xCELLigence (*b*). *κ*_1_ shows a decreasing trend from medium to high antigen levels (*c*) suggesting that high levels of antigen expression may not result in faster rates of CAR T-cell killing. *κ*_2_ increases from low to medium antigen expression and plateaus with high antigen levels (*d*). This suggests limited activation of CAR T-cells at lower antigen expression and that exhaustion rates from medium to high antigen may not change significantly. (Online version in colour.)

Tumour growth rate *ρ* varies significantly (*p* < 0.01) among different cell lines and with antigen expression level (see electronic supplementary material S2 and figure S11a). To investigate the relationship between tumour growth rate and CAR T-cell killing *κ*_1_ and exhaustion *κ*_2_, we evaluated cell lines with antigen levels greater than 80% and treated with BBζ CAR T-cells at an effector to target ratio of 1 : 5. No significant correlation was found between the cancer cell proliferation rate *ρ* and killing rate (*κ*_1_) (electronic supplementary material, figure S11b). However, the exhaustion rate *κ*_2_ is significantly correlated with tumour growth rate (electronic supplementary material, figure S11c) with Pearson correlation coefficient r=−0.9, *p* < 0.001. Similar results were observed for the cells treated with 28ζ IL13Rα2-CARs. Figure 5 shows the density of IL13Rα2 level on cancer cell surface and its relation to CAR T-cell killing for cell line HT1080-H and PBT138-H. We observed that *κ*_1_ shows a decreasing trend from medium to high antigen level (figure 5*c*) suggesting that high levels of antigen expression may not result in faster rates of CAR T-cell killing. The rate constant *κ*_2_ increases from low to medium antigen expression and plateaus with high levels (figure 5*d*). This suggests limited activation of CAR T-cell at lower antigen expression and exhaustion rate from medium to high antigen may not change significantly and may be the result of over-activation of the CAR T-cells.

### 4.5. CARRGO model applied to *in vivo* human data

**Figure 6.**
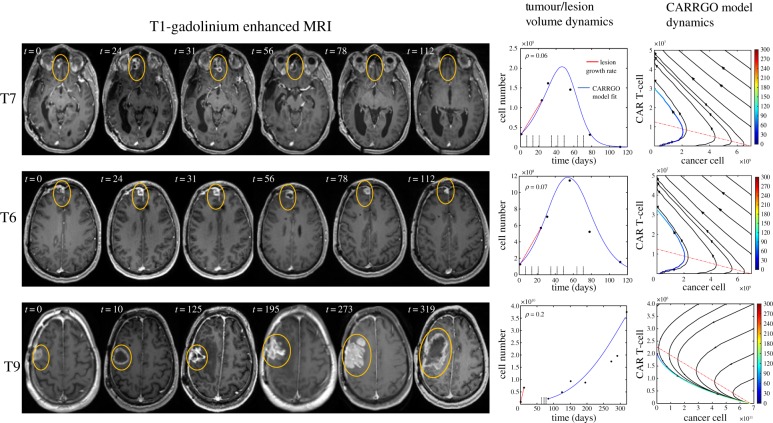
CARRGO model applied to *in vivo* human data. A male patient with multi-focal glioblastoma was treated with IL13Rα2 CAR T-cells. Yellow circles are used to indicate tumour location and do not reflect tumour size. Cell number is calculated from tumour size with volumetric segmentation of the contrast-enhancing lesion. The right columns show CARRGO model fits and dynamics based on the tumour volume data. The CARRGO model parameters are the same for lesions T7 and T6, which responded to CAR T-cell treatment. The model predicts that the non-responding lesion T9 had a smaller rate of CAR T-cell killing and increased rates of exhaustion and CAR T-cell death. Lesion T9 was also observed to have a higher cancer cell proliferation rate (*ρ* = 0.2 day^−1^) when compared with T6 and T7 which had very similar rates (*ρ* = 0.06 and 0.07 day^−1^, respectively). (Online version in colour.)

To translate the *in vitro* dynamics of the model to clinical data [23], we fit the CARRGO model to MRI-derived tumour volumes during CAR T-cell treatment (figure 6). The CARRGO model is able to fit the tumour growth dynamics quite accurately for lesions T6 and T7 with the same set of parameters $\kappa_{1}=6\times 10^{-9}$(day^−1^ cell^−1^), $\kappa_{2}=0.3\times 10^{-10}$ (day^−1^ cell^−1^), $\theta =0.1\times 10^{-5}$(day^−1^) and lesion T9 with $\kappa_{1}=9\times 10^{-8}$(day^−1^ cell^−1^), $\kappa_{2}=-2\times 10^{-13}$ (day^−1^ cell^−1^), $\theta =5\times 10^{-5}$ (day^−1^). In the case of lesion T9, although the CARRGO model is consistent with the overall tumour dynamics, it does not fit the later time points following CAR T-cell treatment well. This is because lesion T9 received radiation treatment between day 200 and 300, which is not included in the CARRGO model. We note the negative correlation between the tumour growth rate (ρ=0.06,0.07 and 0.2 day^−1^ for T6, T7 and T9, respectively) with the CAR T-cell exhaustion rate *κ*_2_ in the patient data, which is consistent with that observed in the experimental data (electronic supplementary material, figure S11c). We remark that the parameters *κ*_1_ and *κ*_2_ are of the order of $O(10^{-13})$, which appear to be very small; however, these parameters are scaled by the carrying capacity *K* in units of cells, which is of order $O(10^{9})$. Therefore, these parameter values are comparable with the *in vitro* data when scaled relative to the carrying capacity (figure 4).

## 5. Discussion

The CARRGO model is a simple representation of cancer cell–T-cell interactions. We developed the CARRGO model with the aim of understanding CAR T-cell efficacy in terms of rates of killing, proliferation, exhaustion, and persistence with a real-time cell analyser in a simple, controlled *in vitro* system. The CARRGO model fitted remarkably well to the highly temporally resolved experimental data and as well as to data derived from a patient treated with IL13Rα2 BBζ CAR T-cells. Although the predator–prey mathematical model formalism has been widely used in a number of biological settings, the novelty of this model is in the application to a novel form of cancer therapy with a high temporal resolution cell monitoring experimental design which provides nearly continuous data on killing kinetics.

With the CARRGO model we show that the rate of cancer cell killing by CAR T-cells is inversely related to the CAR T-cell dose. With a fixed number of cancer cells as an initial condition, as the number of CAR T-cells increases (dose), any individual T-cell will encounter fewer number of cancer cells to kill, indicating that increasing dose does not result in a maximal rate of killing on a per T-cell basis. For example, the PBT138 cell line shows complete killing within 80 and 100 h for effector to target ratios 1 : 5 and 1 : 10, respectively. This result suggests that a lower dose of CAR T-cells may change the time to complete cancer cell killing but shows the same overall cancer cell killing effectiveness. Moreover, we observed that *κ*_2_ positively correlated with CAR T-cell dose. Because the parameter *κ*_2_ is a net measure of CAR T-cell proliferation and exhaustion or lack of activation and the T-cell proliferation is not dose-dependent [18], the trend observed in *κ*_2_ with dose is dominated by the exhaustion rate: the higher the dose, the lower the exhaustion rate, resulting in an increased value of *κ*_2_. The death rate of CAR T-cells was very small when compared with the cancer cell proliferation rate for all conditions. This is likely due to the short time scale of the experiment and because the T-cells were stimulated to proliferate by the presence of cancer cells.

We observed that the cancer cell growth showed no relation with CAR T-cell killing rate and an inverse relationship with *κ*_2_. This may explain variations in patient-specific responses even for the same CAR T-cell dose. For a fixed CAR T-dose, *κ*_2_ is the principal determinant of treatment failure or success as shown in phase-plane analysis (figure 1), which is also observed in patient data (figure 6). This result, driven by the CARRGO model analysis suggests that the balance between proliferation and exhaustion of CAR T-cells may contribute more than the rate of CAR T-cell mediated cancer cell killing in determining treatment success or failure. Moreover, the CARRGO model predicts transient progression of cancer cells even in the case of successful CAR T-cell therapy. This prediction may be consistent with the clinical phenomenon of pseudo-progression, in which the cancer is seen to progress during therapy before eventually responding [7,8]. Identifying characteristics of the patient and the CAR T-cells which may result in pseudo-progression could have a profound effect on interpretation of these dynamics observed in the clinic.

Interestingly, we found *κ*_1_ decreases and *κ*_2_ plateaued from medium antigen level to higher level of antigen expression. One of the possible explanations of this behaviour could be the antigen density is more heterogeneous in the higher antigen level cell population when compared with medium and low antigen levels (figure 5*a*). More heterogeneity in the density of antigen expression intensity in the cancer cells within the initial population may cause clustering of CAR T-cells resulting in their exhaustion [20,40]. Another confounding factor can be the dependence of the detected antigen signal intensity on both the number of antigen-positive tumour cells and their individual antigen expression intensity. Elucidating these factors individually can better tune the model parameters and the prediction of the tumour response dynamics. However, more studies are required to examine effect of cell and population level antigen density on CAR T-cell killing kinetics.

There are some important limitations to consider with this model and experimental system. Perhaps the most obvious is that the *in vitro* system is not a model for the human immune system or tumour microenvironment. It does not include cytokines, stromal cells or additional immune cells such as myeloid cells which contribute to CAR T-cell activity *in vivo*. Another limitation is the assumption that the populations are well mixed. In practice, this assumption may depend on the route of CAR T-cell administration, with intracavitary and intraventricular injections potentially resulting in spatially heterogeneous densities of CAR T-cells or cancer cells, although methods to assess the distribution of CAR T-cells *in vivo* remain an open challenge [41,42]. To address this limitation, the well-mixed assumption may be relaxed and CAR T-cell killing dynamics interrogated with spatial or agent-based models [43]. Another limitation is with regard to the experimental system: the change in electrical impedance measured by the cell index does not differentiate cell detachment from cell killing. This is only a minor consideration as the cell lines used are very adherent to the plate and were not observed to detach. Finally, the experimental system does not directly measure dynamics of CAR T-cells. However, our model is initialized with known numbers of cancer cells and CAR T-cells and the model-predicted cancer cell to CAR T-cell ratio at the experimental endpoint was validated with flow cytometry, giving confidence to our model predictions and parameter estimates. To address this limitation, the CARRGO model CAR T-cell dynamics can be validated by labelling the CAR T-cells and directly measuring their dynamics with live cell imaging-based methods [44]. Despite these limitations, the CARRGO model succeeded in revealing nonlinear dynamics, quantifying kinetics of killing, and generating hypotheses which may be tested in other *in vitro* systems, and other computational or *in vivo* models.

An interesting application of this model would be to adapt the system to evaluate differences between donor T-cells—either autologous or allogeneic. The intrinsic fitness of the T-cell used for CAR T-cell manufacturing is known to be a critical differentiator between responding and non-responding patients [45], and this model may be able to predict T-cell products with high proliferative potential versus products more prone to exhaustion. While our studies used an allogeneic co-culture platform for the predator–prey modelling of effector activity, we expect these findings to be translatable to autologous CAR T-cell therapies, as CAR T-cells recognize targets in an MHC-independent manner. Future studies could specifically evaluate allogeneic and autologous donor-dependent differences to assess the fit of the mathematical model, and guide potential adjustments to the model. The ability to differentiate key quality attributes of the therapeutic product could potentially provide powerful predictions of CAR T-cell product efficacy and facilitate patient treatment management.

In summary, CAR T-cells have shown promise in hematologic malignancies and are being actively investigated in solid tumours. We aimed to use mathematical modelling to investigate factors which contribute to the kinetics of CAR T-cell mediated cancer cell killing in a simple isolated *in vitro* system. We were able to fit the CARRGO model to *in vitro* and *in vivo* human data with remarkable accuracy. We demonstrated that we can consistently and reproducibly estimate rate constants in the CARRGO model and investigate their dependence on CAR T-cell dose and antigen expression levels. The CARRGO model may be combined with other mathematical models which estimate cancer cell growth and proliferation rates non-invasively with MRI data [9,11,46] to produce a fine-tuned and benchmarked suite of mathematical models, which may aide in optimization of dosing and scheduling of CAR T-cells for greater individualized and personalized therapy.

## Supplementary Material

Supplementary data 2 Figs.S8 - S11

## Supplementary Material

Supplementary data 1 Figs S1 - S7

## Supplementary Material

Supplementary Materials Structural indentifibility

## Supplementary Material

Movie S1

## Supplementary Material

Table S1

## Supplementary Material

HT1080

## Supplementary Material

PBT138-high-medium-low

## Supplementary Material

PBT138-030-VeryHigh

## Data accessibility

All raw experimental data are included in the electronic supplementary material.
